# Immune Reconstitution Inflammatory Syndrome Reaction in Patient on Long-Term Prednisone

**DOI:** 10.7759/cureus.38506

**Published:** 2023-05-03

**Authors:** Savina Sahgal, Shivanjali Shankaran, David A Ansell

**Affiliations:** 1 Medical School, Rush University Medical Center, Chicago, USA; 2 Infectious Diseases, Rush University Medical Center, Chicago, USA; 3 Internal Medicine, Rush University Medical Center, Chicago, USA

**Keywords:** autoimmune, prednisone, inflammatory reaction, corticosteroids, iris, tuberculosis

## Abstract

Immune reconstitution inflammatory syndrome (IRIS) can be triggered in many ways. IRIS has been recognized during tuberculosis (TB) therapy, especially in patients newly initiated on antiretroviral therapy for HIV or those taken off immunosuppressives such as tumor necrosis factor-alpha inhibitors. However, there are still many triggers of IRIS that are less understood. This case report describes a patient with scrofula that was concerning for TB reactivation, who then had subsequent IRIS. The patient had been consistently using low-dose long-term prednisone for suppression of his polymyalgia rheumatica. It is suspected that the IRIS reaction could be due to an interaction between rifampin and prednisone causing decreased efficacy of its immunosuppressive effects.

## Introduction

Immune reconstitution inflammatory syndrome (IRIS) is a condition that occurs after the rapid recovery of a compromised immune system that can lead to an excessive and unregulated inflammatory response [1]. IRIS can be classified in different ways, including unmasking IRIS, which may reveal symptoms of a previously asymptomatic infection, and paradoxical IRIS, which is a recurrence of symptoms from a previous infection. The syndrome itself was first identified in patients with tuberculosis (TB) and leprosy, who had a subsequent worsening of symptoms after treatment; however, IRIS is most well studied in the setting of TB-HIV co-infection in patients who are started on antiretroviral (ART) therapy [2,3]. In patients with concomitant TB-HIV, immune system reconstitution from ART therapy can lead to a severe pro-inflammatory reaction and worsening of TB symptoms.

TB-IRIS is currently defined as the initial improvement of TB symptoms and subsequent deterioration, with no other known cause or underlying reason for the reduced efficacy of the TB medication regimen [4]. This deterioration can occur within a matter of weeks.

While HIV may be the most well-known immunocompromised state associated with IRIS, there are other instances in which this reaction may occur. This list specifically includes solid organ transplant recipients, postpartum women, neutropenic patients, and patients receiving tumor necrosis factor-alpha (TNF-alpha) inhibitors such as infliximab and adalimumab [5]. In these instances, patients are initially immunosuppressed, and then some form of immune reconstitution triggers a subsequent IRIS reaction. Patients are then treated with corticosteroids for the management of their inflammatory reaction. However, this case report highlights a unique presentation of suspected TB reactivation and subsequent IRIS in a patient who was already on low-dose prednisone for the management of an autoimmune condition.

## Case presentation

The patient was a 74-year-old Caucasian male with multiple medical conditions, including a history of pulmonary TB (completed treatment in 2019), calcium pyrophosphate disease, and polymyalgia rheumatica, who presented with a new onset mass on his right neck. In 2019, while on adalimumab and prednisone, he was diagnosed with pulmonary TB and completed standard four-drug therapy. Adalimumab was held. He developed fevers, headaches, and confusion one month into treatment, which was thought to be an IRIS presentation, but recovered quickly from this with no intervention. He did well over the next two years.

**Figure 1:**
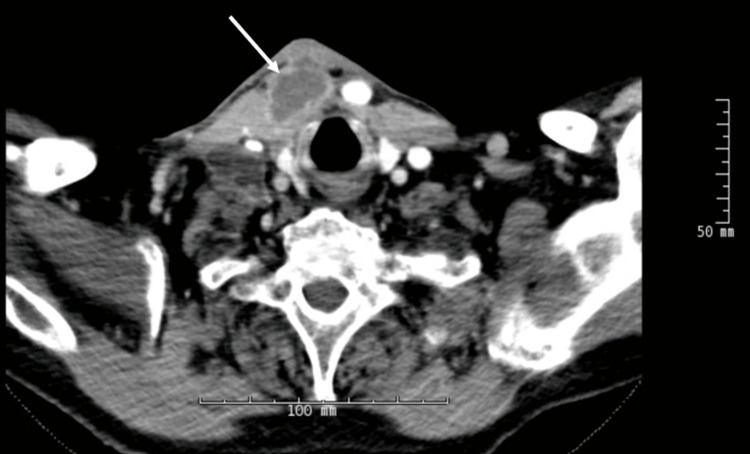
Soft tissue neck CT with IV contrast

**Figure 2:**
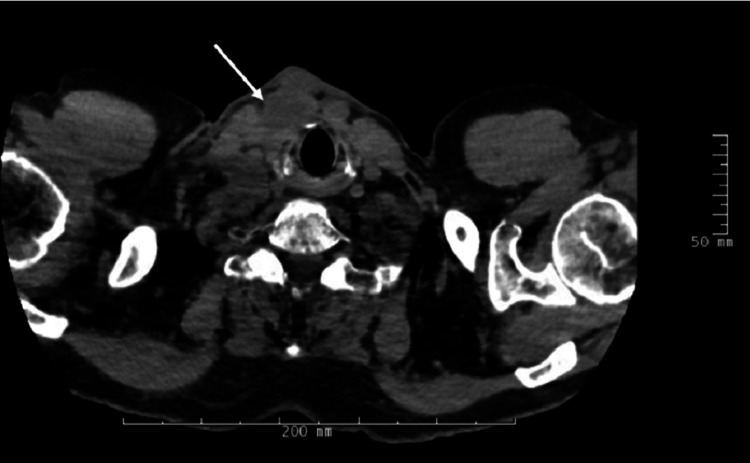
Chest CT without IV contrast

Then he noticed a neck mass one month prior to this most recent presentation associated with discomfort but no other symptoms. Over the course of the month, the mass grew and became red and purulent with associated numbness in his right shoulder due to enlarged axillary lymph nodes. He was on 7 mg of prednisone daily at this time. The patient was admitted for a workup, including a CT soft tissue neck with contrast and a CT chest without contrast, showing cystic lesions in the anterior neck and necrotic cystic lymph nodes (Figures 1, 2).

The anterior cystic lesions were drained and the necrotic lymph nodes were biopsied. Acid-fast bacteria (AFB) smears from the biopsy as well as sputum were positive for *Mycobacterium tuberculosis* (MTB) polymerase chain reaction, which was concerning for a TB reactivation scrofula. Notably, the AFB cultures never grew tuberculosis; however, in the setting of his previous TB infection, he was started on RIPE (rifampin, isoniazid, pyrazinamide, and ethambutol) and linezolid due to concern for multidrug-resistant TB and was discharged. At the time of his TB reactivation, he remained on his 7 mg of prednisone.

**Figure 3:**
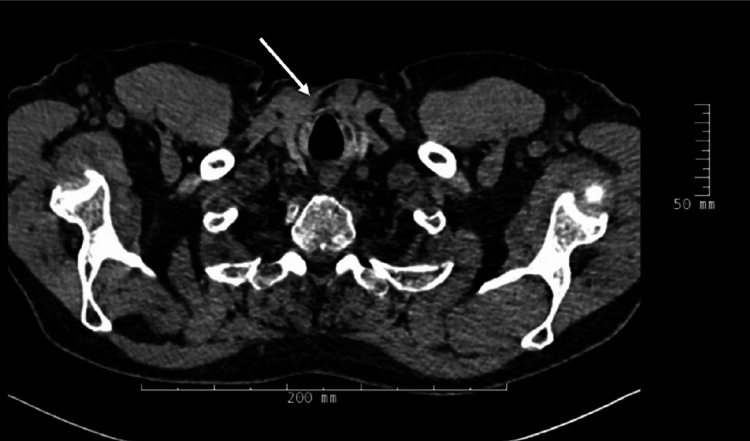
Chest CT without IV contrast after treatment

On RIPE and linezolid, he noticed improvement until about a month into his treatment when symptoms began to worsen, including productive cough, shortness of breath, severe fatigue, brain fog, and significant joint pain and swelling. His symptoms were suspected to be due to IRIS, and this time he was started on a trial dose of prednisone at 60 mg a day. Additionally, rifampin was switched to rifabutin due to the concern that rifampin, as a potent CYP3A4 inducer, may be decreasing the exogenous and endogenous steroid levels leading to adrenal insufficiency. He began to see an improvement on the dose and was started on a slow taper after a couple of weeks of improvement. On repeat CT imaging after completion of his RIPE and linezolid and steroid course, his lymphadenopathy and lung infiltrates had resolved (Figure 3).

## Discussion

This case of TB and then IRIS presents uniquely in a patient with no significant TB reactivation trigger nor a significantly immunosuppressed state that would previously suggest concern for an immune reconstitution reaction. The patient’s initial IRIS presentation in 2019 was one month after the termination of adalimumab, which is a studied trigger of IRIS due to its TNF-alpha inhibitory effects [5]. TNF-alpha is released by macrophages, and macrophages are a primary target of TB. However, he had not taken adalimumab since his initial diagnosis of TB in 2019, and the current understanding is that adalimumab takes approximately six months for complete clearance from the system [6]. There is variability in the exact number of months reported by pharmaceutical companies citing different case reports; however, all suggest the drug is cleared completely in under one year. Our patient has not taken adalimumab in approximately three years.

It is notable that the patient has fluctuated between moderate to low-dose steroids for the last 10 years [7]. It is known that glucocorticoids tend to target lymphocytic cell lines, and that macrophage and monocyte lineages are specifically sensitive to corticosteroid use to lead to immunosuppression [8]. It has also been shown that the half-life and bioavailability of corticosteroids decrease significantly with rifampin [9]. Specifically, respiratory symptoms that require prednisone have been found to be less controlled when a patient is also taking rifampin. Perhaps in the case of this patient, the addition of rifampin, a potent CYP3A4 inducer, may have caused a significant drop in exogenous and endogenous steroids, leading to adrenal insufficiency. This sudden loss of immunosuppression may have also triggered a TB-IRIS. This is further supported by the need to increase the patient’s steroid dosage significantly to control an IRIS reaction, which included notable joint involvement in a patient with polymyalgia rheumatica that was previously controlled on steroids. The patient also only noted significant improvement after changing the rifampin to rifabutin, at which point he was able to tolerate tapering of his steroid dosage. While it is true that the patient never had positive AFB cultures, the CT demonstrating resolution of lymphadenopathy and lung infiltrates after completion of treatment support that this was indeed a TB-IRIS reaction.

## Conclusions

In patients with TB, it is important to consider the potential for IRIS in the setting of reactivation, especially in patients with known autoimmune disorders. While low-dose steroid use is not typically thought to have the same effect as stronger immunosuppressive agents such as TNF-alpha inhibitors, long-term use and subsequent taper can potentially lead to significant immune reconstitution effects. Thus, the taper of immunosuppressive agents should be closely monitored, especially in patients with known TB. Care should also be taken to ensure there is no drug interaction between the immunosuppressant and TB medications.

This case also demonstrates that in the case of TB-IRIS, high-dose steroid use can still be efficacious in controlling the inflammation due to IRIS, but requires replacement of any interacting medications as well as slow and controlled tapering and close monitoring of symptoms.
